# Epidemiology of Influenza-like Illness during Pandemic (H1N1) 2009, New South Wales, Australia

**DOI:** 10.3201/eid1707.101173

**Published:** 2011-07

**Authors:** David J. Muscatello, Margo Barr, Sarah V. Thackway, C. Raina MacIntyre

**Affiliations:** Author affiliations: New South Wales Department of Health, North Sydney, New South Wales, Australia (D.J. Muscatello, M. Barr, S. Thackway);; University of New South Wales, Kensington, New South Wales, Australia (D.J. Muscatello, C.R. MacIntyre);; University of Wollongong, Wollongong, New South Wales, Australia (M. Barr)

**Keywords:** data collection, population, influenza, human, disease outbreaks, viruses, influenza-like illness, Australia, pandemic (H1N1) 2009, research

## Abstract

To rapidly describe the epidemiology of influenza-like illness (ILI) during the 2009 winter epidemic of pandemic (H1N1) 2009 virus in New South Wales, Australia, we used results of a continuous population health survey. During July–September 2009, ILI was experienced by 23% of the population. Among these persons, 51% were unable to undertake normal duties for <3 days, 55% sought care at a general practice, and 5% went to a hospital. Factors independently associated with ILI were younger age, daily smoking, and obesity. Effectiveness of prepandemic seasonal vaccine was ≈20%. The high prevalence of risk factors associated with a substantially increased risk for ILI deserves greater recognition.

During winter 2009, Australia experienced a strong influenza epidemic, caused by the pandemic (H1N1) 2009 virus. In New South Wales (NSW), the most populous state of Australia (≈7 million persons), the epidemic lasted from late June through early September (*1*). Despite intense surveillance and response efforts, determining the epidemiology of influenza at the whole-population level remains difficult, and considerable uncertainty about the disease remains because only a small proportion of infected persons are tested (*2*).

Survey methods have been infrequently used to assess the epidemiology of pandemic influenza virus infection in the general population. In 1919, a personal household interview survey using a sample of population census districts from large population centers was used to assess illness associated with the first wave of pandemic influenza in the Unites States. Persons called intelligent inspectors determined whether the household member was “sick since September 1, 1918, with influenza, pneumonia, or indefinitely diagnosed illness suspected to be influenza.” The survey demonstrated substantial demographic, geographic, and socioeconomic variation in the apparent attack rate of influenza. Expressed as a percentage, the overall incidence rate of clinical infection was estimated to be ≈28% during the first wave. The incidence rate was ≈35% in children and declined with age to ≈30% in adults <35 years of age and to ≈10% in persons >75 years of age. Incidence was higher among women 15–35 years of age than among men in the same age group (*3*,*4*).

During the first epidemic wave of pandemic (H1N1) 2009, we used a continuous population health survey to better understand the epidemiology of the influenza (H1N1) virus in the general community. This situation also created an unprecedented opportunity to assess the prevalence of seasonal influenza vaccination among persons of all age groups in our population and its effectiveness against ILI during pandemic (H1N1) 2009.

## Methods

Since 2002, the NSW Population Health Survey has been operating continuously to provide monthly estimates of health status and risk factors. The survey involves computer-assisted telephone interviews of a randomly selected member of randomly selected households. The target population is state residents in private households with private telephones. The sample is selected by using telephone number ranges obtained from an electronic telephone book that has been geocoded (spatial coordinates assigned to addresses) and stratified by 8 regional health service boundaries within the state. List-assisted random-digit dialing is then used to contact households. The target sample is ≈1,500 persons per regional health service per year, which equals 12,000 persons per year for the state. The survey covers all age groups, and interviews for children <16 years of age are reported by a parent or caregiver. Full details of sample selection and procedures are provided by Barr et al. (*5*). The survey has been approved by the NSW Population Health and Health Services Research Ethics Committee.

When circulation of pandemic (H1N1) 2009 virus in NSW became apparent, ethics approval was obtained to add supplementary questions to the survey to determine the incidence of influenza–like illness in the population and associated health care–seeking behavior and absence from normal duties; these questions were added on July 19, 2009. The questions were as follows: “In the last 4 weeks, did you have an illness with any of the following symptoms: fever or high temperature, cough, sore throat, runny nose, fatigue, chills or shakes, body aches and pains, shortness of breath, the flu or flu–like symptoms?” Responses were recorded for each sign or symptom. Respondents answering “yes” to any sign or symptom were asked the following: “Did you see a GP for this illness?”; “Did you go to a hospital or emergency department for this illness?”; and “How many days were you unable to work, study, or manage day-to-day activities because of the illness?” At the same time, we extended the age range for respondents routinely asked whether they had been “vaccinated or immunized against flu in the past 12 months” to persons >6 months of age. Previously, the question had been asked only of persons >50 years of age. The extended age range would enable assessment of vaccine effectiveness against ILI.

We defined ILI as self–reported fever or high temperature with cough and fatigue. In a range of general practice surveillance systems for seasonal influenza in Australia, this definition performed better than alternative definitions; positive predictive value was 23%–60% and negative predictive value was 64%–91% (*6*).

To obtain monthly estimates of ILI incidence, for any respondents reporting a symptom in the past 4 weeks we assigned their illness to the middle of the reference period, that is, 14 days before the interview date. This illness date was then assigned to a month of illness.

Answers to other questions routinely asked in the survey enabled analysis of additional factors that might be associated with ILI reporting (*7*), including age (0–15, 16–34, 35–49, 50–64, or >65 years); sex; household size (1–2 or >3 residents); number of children in household (<2 or >2); urban or rural location of residence; socioeconomic disadvantage at respondent’s residential postal code, which is derived from the Australian Census and takes into account income, education, occupation, employment status, indigenous status, housing, and other variables (index of relative socioeconomic disadvantage [*8*]: lowest 2 quintiles = disadvantaged and upper 3 quintiles = not disadvantaged); current asthma (respondents >2 years of age, diagnosis made by a doctor, and symptoms or treatment in the past 12 months); nongestational diabetes or high blood glucose status (>9 years, diagnosis made by a doctor); smoking status of persons >16 years of age (daily smoker, occasional smoker, ex-smoker, or nonsmoker); body mass index ([BMI] [weight in kilograms]/[height in meters]^2^) of persons >2 years of age (>30 = obese, 25 to <30 = overweight, and <25 = healthy or underweight); alcohol drinking at levels associated with health risk (>2 Australian standard drinks [1 standard drink = 10 g alcohol] on any day) (*9*); adequate physical activity for persons >16 years of age (at least 150 minutes exercising per week on >5 occasions, adequate or not adequate), psychological distress score for persons >16 years of age (Kessler–10 scale ([*10*] high or very high [score >22], moderate, or low); and vaccinated against pneumococcal disease in the past 5 years for persons >50 years of age (yes or no).

As is standard for household population surveys, the data record for each survey respondent was assigned a numeric weighting, which was used in all analyses to scale their results to the total NSW population. The weighting value takes into account the probability (by age, sex, and geographic region) of being selected for participation in the survey (*5*). Regression models were used to obtain the relative risk (RR) of reporting ILI for each of the factors listed in the previous paragraph. The dependent variable for each model was ILI, which was assigned 1 of 2 values: 1 if the respondent met the criteria for ILI and 0 otherwise. The independent variables were >1 factor.

Our modeling strategy was to individually test the association between each factor and ILI by using a regression model and then to develop a final model incorporating multiple factors to assess whether independent associations remained. Because age was strongly associated with ILI, we included it as an independent variable in the model for all single-factor assessments. Factors with p<0.1 for an individual association were included in the final model. Despite its nonsignificance, sex was included in the final model because the prevalence of risk factors in our population was known to differ by sex.

To estimate RRs from survey data instead of the more usual odds ratios, we used Poisson regression analysis with robust variance estimation. RRs were calculated by using the GENMOD procedure included in SAS Statistical Analysis Software version 9.1.3 for Windows (Cary, NC, USA) with the following programming statements: a model statement with options dist = Poisson and link = log; a class statement including the unique survey respondent number variable; a repeated statement with an independent correlation structure (corr = ind) and specifying the unique survey respondent number variable as the subject parameter; and a weight statement specifying the respondent sample weighting normalized to sum to the total sample size (*11*–*13*). Because the Poisson model uses the natural logarithm as the link function, exponentiation of the parameter estimates was used to obtain the RR for the study factors.

Vaccine effectiveness for ILI was estimated by 1–RR. RR was the age-adjusted relative risk of reporting ILI among respondents reporting seasonal influenza vaccination in the past 12 months relative to that for unvaccinated respondents. RRs were obtained from the regression analysis (*14*).

## Results

### Incidence of ILI

From July 19 through October 14, 2009, completed interviews were obtained from 2,909 respondents from 5,017 eligible households contacted during that period. Participation rate was 58.0%.

During July 2009, estimated ILI incidence was 12.1% (95% confidence interval [CI] 9.1%–15.0%), representing 850,000 (95% CI 640,000–1,060,000) persons (Table 1). Incidence declined to 7.4% (95% CI 5.3%–9.5%) in August, and 3.6% (95% CI 1.2%–5.9%) in September. Assuming that during the 3-month window each person could only experience ILI 1 time, the monthly incidence can be summed to provide an estimate of the total proportion of the population experiencing ILI during that period. This calculation indicated that an estimated 23.1% (95% CI 18.8%–59.9%) of the NSW population, or 1,630,000 (95% CI 1,330,000–4,240,000) persons, experienced ILI during that period.

**Table 1:** Influenza-like illness cases, by time period, New South Wales, Australia, July–September 2009*

Period	Estimated no., millions	Estimated incidence, % (95% CI)
July	0.85 (0.64–1.06)	12.1 (9.1–15.0)
August	0.52 (0.38–0.67)	7.4 (5.3–9.5)
September	0.25 (0.09–0.42)	3.6 (1.2–5.9)
July–September	1.63 (1.33–1.94)	23.1 (18.8–27.4)

*Data from 2,909 New South Wales Population Health Survey respondents. Influenza-like illness defined as fever with cough and fatigue. CI, confidence interval.

The only significant difference was the low incidence for persons >65 years of age (5.1%; 95% CI 2.4%–7.9%), compared with estimates of 19.1% (95% CI 11.9%–26.2%) for those 50–64 years of age and 33.3% (95% CI 24.7%–42.0%) for those 0–15 years of age (Table 2). Estimates were higher for female than male respondents and for residents of rural areas, but these differences were not significant.

**Table 2:** Influenza–like illness incidence, New South Wales, Australia, July–September 2009*

Group	Incidence, % (95% CI)
Sex	
M	19.5 (13.8–25.2)
F	26.9 (20.4–33.4)
Age group, y	
0–15	33.3 (24.7–42.0)
16–34	27.6 (15.6–39.6)
35–49	21.3 (13.9–28.8)
50–64	19.1 (11.9–26.2)
>65	5.1 (2.4–7.9)
Region	
Urban	22.3 (16.8–27.8)
Rural	26.2 (18.4–34.0)

*Data from 2,909 New South Wales Population Health Survey respondents. Influenza-like illness defined as fever with cough and fatigue. CI, confidence interval.

### Outcome of ILI

Inability to undertake normal duties for <3 days was reported by approximately half (51%, 95% CI 41%–61%) of those reporting ILI (Table 3). Another 12% (95% CI 6%–18%) were unable to continue their usual duties for at least 7 days. Approximately half (55%) sought care at a general practice (95% CI 46% – 65%), and 5% (95% CI 1%–9%) sought care at a hospital.

**Table 3:** Illness outcomes for persons reported influenza–like illness, New South Wales, Australia, July–September 2009*

Outcome	Estimated no. persons, millions (95% CI)†	Proportion, % (95% CI)
No. days unable to do usual activities		
0–3	0.83 (0.67–1.00)	51.3 (41.4–61.1)
4–7	0.60 (0.44–0.76)	36.7 (26.9–46.5)
>7	0.20 (0.09–0.30)	12.0 (5.7–18.4)
Type of health care sought		
General practice	0.90 (0.74–1.06)	55.4 (45.5-65.2)
Hospital	0.08 (0.02–0.15	5.2 (1.3–9.2)
General practice and hospital‡	0.04 (0.01–0.07	2.3 (0.5–4.1)
Neither general practice nor hospital	0.68 (0.53–0.83)	41.7 (32.3–51.1)

*CI, confidence interval. †The estimated numbers were obtained by applying the proportions and 95% CIs in this table to the estimated 1.63 million persons with influenza–like illness (Table 1). Standard errors of that estimate were ignored. ‡Because this category overlaps the 2 categories above, total is not 100%.

### Prevalence of Seasonal Influenza Vaccination

When we calculated prevalence of seasonal influenza vaccination for all age groups, we found that one quarter (25%, 95% CI 23%–28%) had been vaccinated against influenza in the previous 12 months. The proportion was highest among persons >65 years of age (74%, 95% CI 71%–78%), fell to 33% (95% CI 29%–38%) among those 50–64 years, and was <20% among those <50 years (Table 4). Previous estimates in NSW were based on persons >50 years of age because national immunization policy focused on this age group.

**Table 4:** Prevalence of seasonal influenza vaccination in past 12 months, New South Wales, Australia, July–September 2009*

Group	Prevalence, % (95% CI)
Total NSW population	25.4 (23.3–27.5)
Sex	
M	24.6 (21.3–27.9
F	26.3 (23.7–28.8
Age, y	
0–15	11.5 (7.5–15.5)
16–34	14.8 (10.2–19.3
35–49	16.0 (11.7–20.2
50–64	33.2 (29.0–37.5)
>65	74.3 (70.7–77.9
Region	
Urban	24.7 (22.1–27.3)
Rural	27.2 (23.7–30.7)

*Data from 2,909 total New South Wales Population Health Survey respondents. CI, confidence interval; NSW, New South Wales.

### Factors Associated with Reporting ILI

Younger age was strongly associated with ILI (Table 5), which was ≈6× more likely to be reported for children <15 years of age than for persons >65 years of age. Therefore, for evaluation of all other individual factors, we adjusted for age. Only obesity and daily smoking were positively associated with reporting ILI. All remaining factors showed no significant association. In the final regression model, we included age, sex, BMI, and smoking status. Although sex was not associated with ILI, obesity and smoking in our population varied substantially by sex and were included in the model. Only persons >16 years of age were included because 16 years was the youngest age for which smoking questions were asked.

**Table 5:** Risk factors evaluated for influenza-like illness, New South Wales, Australia, July–September 2009*

Factor	Factor prevalence, % (95% CI)	Reference category†	Age group analyzed (no. respondents)‡	Relative risk (95% CI)	p value§
Age, y		>65 y	All ages (2,909)		
0–15	21.4 (19.4–23.5)			5.96 (3.25–10.93)	**<0.001**
16–34	26.0 (23.2–28.8)			4.76 (2.35–9.62)	**<0.001**
35–49	21.7 (19.4–23.9)			3.55 (1.86–6.79)	**<0.001**
50–64	17.7 (16.1–19.3)			3.00 (1.57–5.71)	**0.001**
Female sex¶	50.5 (47.8–53.2)	Male	All ages (2,909)	1.31 (0.89–1.94)	0.173
Rural region¶	29.9 (28.4–31.4)	Urban	All ages (2,909)	1.26 (0.87–1.84)	0.227
Socioeconomically disadvantaged¶ #	38.4 (35.9–40.9)	Not disadvantaged	All ages (2,909)	0.93 (0.63–1.38)	0.729
>2 children in household¶	34.5 (31.9–37.2)	<2 children residing in household	All ages (2,909)	0.75 (0.51–1.10)	0.142
>3 persons in household¶	67.7 (65.6–69.7)	1–2 persons in household	All ages (2,909)	1.14 (0.75–1.72)	0.536
Seasonal influenza vaccination in past 12 mo¶	25.6 (23.5–27.7)	No seasonal influenza vaccination in past 12 mo	>6 mo (2,888)	0.80 (0.49–1.31)	0.372
Current asthma¶	11.4 (9.8–13.0)	No current asthma	>2 y (2,831)	1.15 (0.69–1.92)	0.595
Body mass index¶			>2 y (2,831)		
Obese	18.0 (15.9–20.1)	Healthy or underweight		2.14 (1.31–3.48)	**0.002**
Overweight	28.6 (26.3–31.0)	Healthy or underweight		1.06 (0.67–1.67)	0.816
Current diabetes or high blood glucose¶	8.0 (6.7–9.2)	No current diabetes or high blood glucose	>9 y (2,636)	0.97 (0.50–1.91)	0.936
Daily smoker¶	13.0 (10.8–15.1)	Occasional/ex-/non-smoker	>16 y (2,431)	1.94 (1.06–3.54)	**0.031**
Inadequate physical activity¶	48.6 (45.6–51.7)	Adequate physical activity	>16 y (2,431)	1.04 (0.65–1.65)	0.881
Risky alcohol drinking¶	31.6 (28.6–34.7)	Low-risk alcohol drinking	>16 y (2,431)	1.25 (0.76–2.05)	0.378
High or very high psychological distress score¶	11.2 (9.4–13.1)	Moderate or low psychological distress score	>16 y (2,431)	1.41 (0.85–2.35)	0.182
Pneumococcal vaccination in past 5 y¶	28.3 (25.7–30.9)	No pneumococcal vaccination in the past 5 y (adjusted for age)	>50 y (1,633)	1.20 (0.56–2.56)	0.640

*CI, confidence interval. †Not stated and “Don’t know” responses were included in the reference category. Among parameters with any such responses, the proportions were index of socioeconomic disadvantage 0.9%, vaccinated against influenza in the past 12 mo 1.0%, current asthma 0.3%, body mass index 5.2%, smoking status 0.1%, psychological distress score 3.0%, alcohol use 1%, and pneumococcal vaccination 7.0%. ‡Some questions are only collected on selected age groups, so sample sizes vary. §Significant results at the 5% level are in **boldface**. ¶Adjusted for age. #Disadvantaged = lowest 2 quintiles of the Australian index of relative socioeconomic disadvantage based on the respondent's residential postcode; not disadvantaged = upper 3 quintiles.

The association with younger age remained in the final model (Table 6). Among the 13.0% (95% CI 10.8%–15.1%) of the population >16 years of age who reported daily smoking, the risk for ILI was 90% (95% CI 10%–226%) higher than that for less frequent smokers and nonsmokers combined. Among the 18.0% (95% CI 15.9%–20.1%) whose BMI was in the obese category, the risk for ILI was 132% (95% CI 30%–316%) greater than that for the combined group of persons whose BMI was healthy or underweight.

**Table 6:** Final model of factors associated with reporting influenza-like illness, New South Wales, Australia, July–September 2009*

Factor, n = 2,431 respondents	Prevalence of factor, % (95% CI)	Reference category†	Relative risk (95% CI)	p value‡
Age, y		>65 y		
16–34	26.0 (23.2–28.8)		4.74 (2.38–9.45)	**<0.001**
35–49	21.7 (19.4–23.9)		3.25 (1.68–6.26)	**<0.001**
50–64	17.7 (16.1–19.3)		2.60 (1.36–4.99)	**0.004**
Female sex	50.5 (47.8–53.2)	Male	1.25 (0.76–2.05)	0.375
Daily smoker	13.0 (10.8–15.1)	Occasional/ex-/nonsmoker	1.90 (1.10–3.26)	**0.021**
Body mass index				
Obese	18.0 (15.9–20.1)	Healthy or underweight	2.32 (1.30–4.16)	**0.005**
Overweight	28.6 (26.3–31.0)	Healthy or underweight	1.20 (0.68–2.12)	0.534

*Influenza-like illness defined as fever with cough and fatigue. Some questions are only collected on selected age groups, so sample sizes vary. CI, confidence interval. †Not stated and “Don’t know” responses were included in the reference category. Among parameters with any such responses, the proportions were body mass index 5.2%; smoking status 0.1%. ‡Statistically significant results at the 5% level are in **boldface.**

### Effectiveness of Seasonal Influenza Vaccine

When the age-adjusted RR for ILI among persons reporting vaccination with the seasonal influenza vaccine in the past 12 months was used, the estimated vaccine effectiveness was 20.0% (95% CI –30.5% to 51.0%), indicating a possibly mild but nonsignificant benefit. Analysis of effectiveness in specific age groups and by sex, region, smoking status, or obesity did not indicate any significant benefit (Table 7).

**Table 7:** Effectiveness of seasonal influenza vaccine against influenza-like illness, New South Wales, Australia, July–September 2009*

Subgroup, n = 2,888	Vaccine effectiveness, % (95% CI)†
Age, y	
0.5–15	6.1 (−120.1 to 60.0)
16–34	44.6 (−166.8 to 88.5)
35–49	−0.9 (−137.2 to 57.1)
50–64	33.7 (−69.7 to 74.1)
>65	−97.9 (−622.5 to 45.8)
Sex†	
M	34.5 (−71.7 to 75.0)
F	10.5 (−49.2 to 46.4)
Region†	
Urban	17.1 (−55.7 to 55.9)
Rural	25.5 (−49.7 to 62.9)
Smoking status†	
Daily smoker	1.4 (−175.3 to 64.7)
Not daily smoker	22.3 (−53.2 to 60.6)
Body mass index†	
Obese	25.0 (−81.6 to 69.1)
Not obese	9.2 (−66.0 to 50.4)
Overall†	20.0 (−30.5 to 51.0)
*For persons >6 mo of age vaccinated in past 12 mo. CI, confidence interval. †Adjusted for age.	

## Discussion

In NSW, during the first Southern Hemisphere winter in which pandemic (H1N1) 2009 virus was circulating, at least one quarter of the population and one third of children experienced ILI. Many infections other than influenza can cause ILI (*15*,*16*); however, this study was conducted during the peak months of the epidemic in NSW, when the predictive value of ILI for influenza infection would be optimal (*6*). The epidemic was recognized in Australia after mid-June and grew rapidly (*1*). We were able to obtain full monthly estimates of ILI from July only. Late June was part of the recall period of the survey questions for respondents interviewed in July, and some of that activity may have been included in the July estimate.

ILI incidence was similar in urban and rural regions and in each sex. Approximately half the persons who reported ILI had to limit their usual activities for <4 days. Approximately half sought care for their illness at a general practice, and 5% sought care at a hospital. Vaccination against seasonal influenza did not protect against ILI. Daily smoking and obesity each independently doubled the risk for ILI.

Consistent with the known epidemiology of pandemic (H1N1) 2009 virus infection (*1*,*17*–*19*), incidence of ILI decreased with age; the decline was sharp for those >65 years of age. The age-specific estimates of ILI incidence in NSW in 2009 were remarkably similar to those reported during the 1918 influenza pandemic in the United States (*4*). Our overall estimate of an ILI rate of 23% was higher than the overall population infection rate for pandemic (H1N1) 2009 of 16% estimated by a recent seroprevalence study from NSW (*20*). Although the CIs of both estimates overlapped and thus the estimates did not differ significantly, explanations for our higher estimate could be as follows: 1) some of the ILI in our study was caused by other influenza strains that circulated earlier in the season (*1*) and by pathogens other than influenza; 2) our study included information collected through the end of September, whereas the seroprevalence study included some specimens collected before the end of the epidemic during August; and 3) population samples differed. However, the seroprevalence study detected mild and asymptomatic infections. Because the seroprevalence study used specimens requested from clinical chemistry laboratories without randomization, statistical biases may arise from the nonrandom sample selection and the disease factors leading to a clinical specimen being required. Age-specific comparisons between the 2 studies were broadly consistent. Serosurvey infection rate estimates were 10%–35% among children, 24% among adults 18–34 years of age, 20% among adults 35–64 years of age, and no infection among older adults. Our estimates for ILI were 33% among children, 28% among persons 16–34 years of age, 20% among adults 35–64 years of age, and 5% among older adults. The seroprevalence study estimated reduced infection rates outside the urban state capital of Sydney; our study found similar incidence rates in urban and rural areas.

Other studies that assessed household size and risk for transmission found mixed results: some found increased risk (*21*,*22*) and another found decreased risk (*23*) with increasing household size. More children in households has also been identified as a risk factor for influenza transmission in households (*24*). However, our finding of no association with either household size or number of children in the household is consistent with the result of household transmission studies of the pandemic 2009 (H1N1) virus (*18*,*25*) and with results of another study of ILI among children during seasonal influenza season in Australia (*26*). This lack of effect may reflect improved living standards in this country.

The lack of protection from recent seasonal influenza vaccination has been reported elsewhere (*18*,*27*,*28*), but a study in Mexico found partial protection (*29*). The subtype H1N1 component of the Northern and Southern Hemisphere vaccines at that time was the same: A/Brisbane/59/2007–like.

Obesity and smoking are 2 preventable risk factors we found to be strongly associated with ILI. Smoking has been frequently identified as a risk factor for influenza; the identified mechanisms are mechanical, structural, and immunity related (*30*–*32*). Although obesity has been frequently identified as a risk factor for severe outcomes of infection with pandemic 2009 (H1N1) virus (*33*–*35*), it has not been previously recognized as a risk factor for susceptibility to symptomatic influenza infection in humans. A recent study in mice found that an immune memory response to recent influenza infection was reduced among obese mice; this reduced memory led to more severe disease, lung pathology, and virus titers after a second exposure to the same mouse-adapted influenza strain (*36*).

In addition to possibly excluding the early part of the epidemic, our study has other limitations. Influenza in respondents was not confirmed by testing; other common winter respiratory viruses, such as respiratory syncytial virus, can cause a similar syndrome (*16*). General practice surveillance in various regions of Australia, conducted during circulation of seasonal influenza virus, indicated that the syndrome definition we used had a positive predictive value of 23%–60% (*6*). Although these values are not high, positive predictive value is probably increased during a larger than usual epidemic (*37*). Pandemic concern may have prompted more persons than usual to get vaccinated for seasonal influenza. This concern and response would produce higher vaccination prevalence in our study than would have occurred in the absence of a pandemic. Evidence shows that publicity prompted increased vaccination among persons >65 years of age, from 68% in April 2009 to 77% in May 2009. Prevalence remained higher for several months (*38*). In our study, we were unable to include 2 frequently reported risk factors for poor outcomes of pandemic (H1N1) 2009 virus infection: pregnancy and indigenous status (*39*,*40*). Although indigenous status is included in the health survey, the number of Aboriginal and pregnant respondents in the period of time covered would be too small to obtain usable estimates for these risk factors.

## Conclusions

When pandemic (H1N1) 2009 virus was circulating in the NSW population, ILI was experienced by at least one quarter of the population. Recent prepandemic seasonal vaccination was not protective. Although smoking is already known to increase susceptibility to influenza infection, obesity is not. The role of obesity in susceptibility needs further evaluation in studies in which influenza infection can be confirmed. The high prevalence of these preventable risk factors in our population, combined with a substantially increased risk for ILI, deserves greater recognition. Using an established health survey for monitoring ILI is inexpensive and provides an opportunity to assess a broad range of risk factors. Continued monitoring will enable better assessment of the value of survey-based influenza surveillance through comparison with other influenza and respiratory illness surveillance systems and can provide continuous assessment of risk factors for ILI.

## Appendix

34. ANZIC Influenza Investigators, Webb SA, Pettilä V, Seppelt I, Bellomo R, Bailey M,  Critical care services and 2009 H1N1 influenza in Australia and New Zealand. [**PMID: 19815860**]. N Engl J Med. 2009;361:1925–34. Epub 2009 Oct 8. 10.1056/NEJMoa090848119815860
